# Essential Oils with High Activity against Stationary Phase *Bartonella henselae*

**DOI:** 10.3390/antibiotics8040246

**Published:** 2019-11-30

**Authors:** Xiao Ma, Wanliang Shi, Ying Zhang

**Affiliations:** Department of Molecular Microbiology and Immunology, Bloomberg School of Public Health, Johns Hopkins University, Baltimore, MD 21205, USA; xma37@jhmi.edu (X.M.); wshi3@jhu.edu (W.S.)

**Keywords:** Essential oils, *Bartonella henselae*, persisters, stationary phase, antimicrobial activity

## Abstract

*Bartonella henselae* is a fastidious Gram-negative intracellular bacterium that can cause cat scratch disease, endocarditis in humans and animals, as well as other complications, leading to acute or chronic infections. The current treatment for *Bartonella* infections is not very effective presumably due to bacterial persistence. To develop better therapies for persistent and chronic *Bartonella* infections, in this study, with the help of SYBR Green I/PI viability assay, we performed a high-throughput screening of an essential oil library against the stationary phase *B. henselae*. We successfully identified 32 essential oils that had high activity, including four essential oils extracted from *Citrus* plants, three from *Origanum*, three from *Cinnamomum*, two from *Pelargonium,* and two from *Melaleuca*, as well as frankincense, ylang-ylang, fir needle, mountain savory (winter), citronella, spearmint, elemi, vetiver, clove bud, allspice, and cedarwood essential oils. The minimal inhibitory concentration (MIC) determination of these 32 top hits indicated they were not only active against stationary phase non-growing *B. henselae* but also had good activity against log-phase growing *B. henselae*. The time-kill assay showed 13 active hits, including essential oils of oregano, cinnamon bark, mountain savory (winter), cinnamon leaf, geranium, clove bud, allspice, geranium bourbon, ylang-ylang, citronella, elemi, and vetiver, could eradicate all stationary phase *B. henselae* cells within seven days at the concentration of 0.032% (*v/v*). Two active ingredients, carvacrol and cinnamaldehyde, of oregano and cinnamon bark essential oils, respectively, were shown to be very active against the stationary phase *B. henselae* such that they were able to eradicate all the bacterial cells even at the concentration ≤ 0.01% (*v/v*). More studies are needed to identify the active components of some potent essential oils, decode their antimicrobial mechanisms, and evaluate their activity against *Bartonella* infections in animal models.

## 1. Introduction

*Bartonella* species are fastidious, Gram-negative, facultative intracellular pathogens [1,2,3] that can be transmitted to humans or animals by several arthropod vectors including fleas, sheep keds, lice, sand flies, ticks, and potentially mites and spiders. *Bartonella* bacteria can infect healthy people while being considered especially important as opportunistic pathogens [4]. At least 13 *Bartonella* species are known to be able to infect humans, causing either acute or chronic infections which could lead to cat scratch disease, endocarditis, bacillary angiomatosis [3], bacteremia and central nervous system pathologies [5]. This pathogenicity is partly due to their unique infection cycle including the lymphatic stage [6] and intraerythrocytic stage [7,8]. It is laborious using classical bacteriological methods to isolate and culture *Bartonella* spp. in liquid media especially from clinical samples, which requires specific conditions and prolonged incubation periods [9,10]. Therefore, serology and real-time PCR are often used instead of culture to confirm the diagnosis for rapid *Bartonella* detection clinically [9,10]. The first-line antibiotics for treating bartonellosis include doxycycline, erythromycin, gentamicin, rifampicin, azithromycin, and ciprofloxacin, as well as some drug combinations like doxycycline plus gentamicin, doxycycline plus rifampin [11,12]. However, a systematic review has revealed that the current clinical treatment of *Bartonella* infections relies mostly on personal experience, expert opinion, and microbiological susceptibility data. The treatment lacks evidence of randomized trials and the recommended antibiotic treatment for cat scratch disease, infectious endocarditis, and bacillary angiomatosis showed no improvement in cure rate or cure time [13]. In particular, there is no single treatment effective for systemic *B. henselae* infections, and antibiotic therapy exhibited poor activity against typical uncomplicated cat scratch disease [11,12]. Therefore, bartonellosis treatment remains a significant problem and without treatment, it could cause high mortality in some patients. The phenomenon of bacterial persistence may partly contribute to the difficulty to treat the disease because persister bacteria are hard to be eradicated and remain in the host which can revert to growing forms under appropriate conditions and lead to relapse or prolonged infections with symptoms [14]. Thus, identifying drugs that target *Bartonella* persister cells in the stationary phase could provide a promising strategy for developing a more effective treatment for bartonellosis. In our recent study, we have identified some promising drug candidates from the FDA drug library that are more active than the current drugs used to treat *Bartonella* infections. However, their utility remains to be further validated.

The essential oil, also known as volatile oil or ethereal oil, is a concentrated hydrophobic liquid containing volatile chemical compounds extracted from plants. It has many uses in aromatherapy [15], food processing [16], and also potentially in medical therapy [17] especially with recent concerns about antibiotic resistance. Previous in vitro studies have found certain essential oils had antibacterial activity against multidrug-resistant Gram-negative clinical isolates [18]. In fact, many essential oil compositions including carvacrol, thymol, cinnamic acid, trans-cinnamaldehyde, eugenol, α-pinene, and γ-terpinene have been documented to have antimicrobial activities since decades ago, and some essential oil components could have a synergistic effect in combination with antibiotics [19]. We have previously used a rapid high-throughput drug screening method using SYBR Green I/PI viability assay [20] for the successful identification of many essential oils with high activity against stationary phase *Borrelia burgdorferi* [21,22] as a surrogate model of persister bacteria [23]. In this study, we adapted the same SYBR Green I/PI methodology to perform an efficient screen using our essential oil collection against stationary phase *B. henselae* and identified a significant number of essential oils that had good activity against non-growing *B. henselae* cells. The implication of the identified active hits for improved treatment of persistent *Bartonella* infections is discussed.

## 2. Results

### 2.1. Subsection Screening Essential Oil Collection to Identify Drugs Active against Non-Growing Stationary Phase B. henselae

Previously, we have developed an SYBR Green I/PI viability assay for the rapid viability assessment of *B. henselae* and have successfully used this assay for high-throughput drug screens against non-growing stationary phase *B. henselae* using the FDA drug library [24]. Here we adapted this SYBR Green I/PI viability assay for essential oil screens against *B. henselae*. As described in the previous study, a five-day-old stationary phase *B. henselae* culture was used to identify active essential oils against stationary phase *B. henselae*. All 149 essential oils were applied at two concentrations, 0.5% (*v/v*) and 0.25% (*v/v*), respectively, in the primary screens. Meanwhile, the currently known effective antibiotics used to treat bartonellosis such as doxycycline, azithromycin, gentamicin, rifampin, etc. were included as control drugs for comparison (Table 1). In addition, we included previously identified FDA-approved drugs that were effective against *B. henselae* such as daptomycin, methylene blue, miconazole and nitrofurantoin [24] as controls (Table 1). All these antibiotics were used at 20 μM. In the primary screens, 32 of the 149 essential oil collection were found to have good activity against stationary phase *B. henselae* both at the concentration of 0.5% and 0.25%, and thus were selected as top hits. The top 32 active hits were chosen based on their lower percentage of viable cells remaining after essential oil treatment than that for the current antibiotics used to treat *Bartonella* infections, including doxycycline, gentamicin, moxifloxacin, azithromycin, and rifampin. According to our previous experience, some compounds in the essential oils can cause interference with the SYBR Green I/PI assay because of color and autofluorescence. Thus, we selected these 32 top hits for further validation by microscopic counting to confirm the SYBR Green I/PI plate reader results. The currently used antibiotics for bartonellosis treatment and the identified FDA-approved drugs effective against *B. henselae* were also included as controls for comparison at 20 μM. Doxycycline as a control drug showed mild activity against stationary phase *B. henselae* (residual viability above 26%) (Table 1). Antibiotics reported to have a clinical improvement for *Bartonella* infection including moxifloxacin, gentamicin, azithromycin, and rifampin [25,26] showed relatively better activity (residual viability between 9% and 25%) against stationary phase *B. henselae* than doxycycline. FDA-approved drugs that we identified as effective against stationary phase *B. henselae* (daptomycin, methylene blue, miconazole, and nitrofurantoin) had better activity (residual viability between 8% and 19%) than most of the five antibiotics mentioned above.

**Figure 1:**
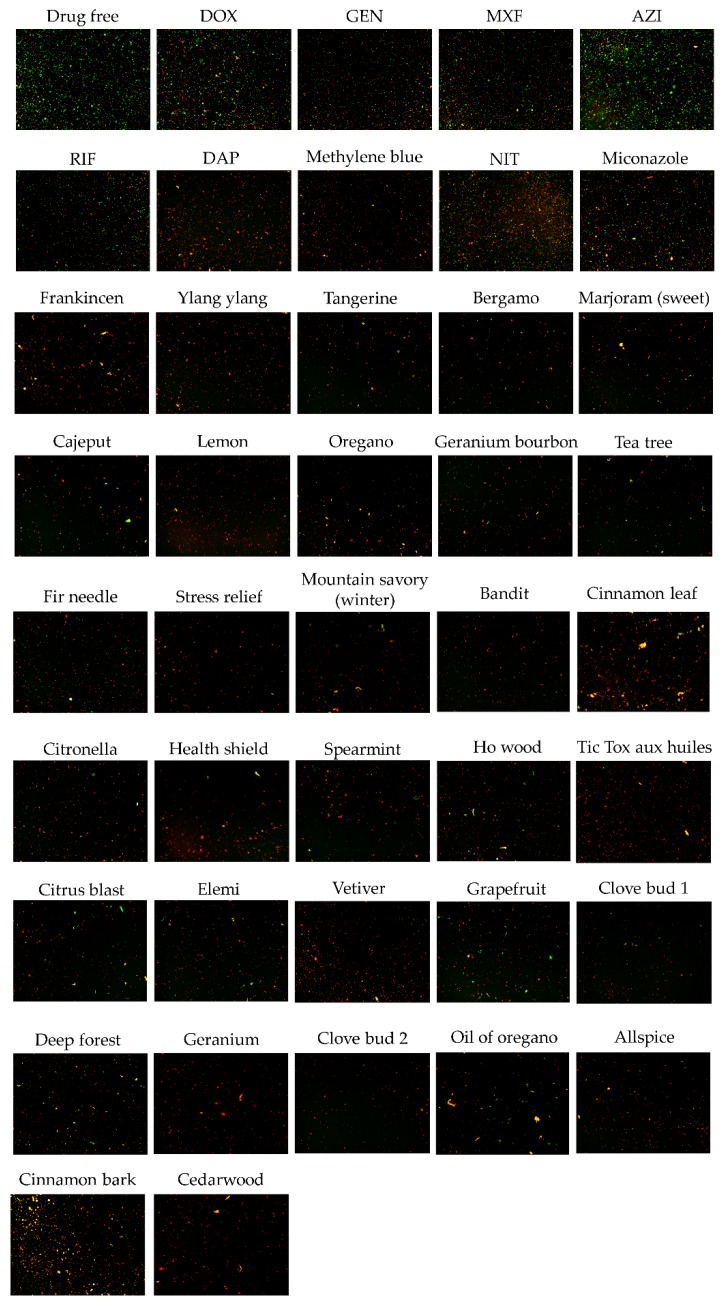
Effect of 32 top hits of essential oils against stationary phase *B. henselae* JK53 in comparison with control drugs. A five-day-old stationary phase *B. henselae* JK53 culture was treated with 0.25% (*v/v*) essential oils or control drugs (20 μM) for three days followed by SYBR Green I/PI viability assay and fluorescence microscopy.

Among the 32 top hits that had better activity (residual viability between 5% and 21%) against stationary phase *B. henselae* than most control antibiotics, the most active essential oils were ylang-ylang, lemon, stress relief, health shield, Tic Tox aux huiles essentielles, geranium essential oil, clove bud, and cedarwood because of their remarkable activity at 0.25%, as shown by red (dead) cells in fluorescence microscope observation (Figure 1). Essential oils made from oregano (“oregano” and “oil of oregano”) and cinnamon (“cinnamon leaf” and “cinnamon bark”) were all shown to be active against stationary phase *B. henselae*, which have already been identified effective against stationary phase *B. burgdorferi* in our previous study [21]. Some essential oils extracted from plants of the same genus as oregano or cinnamon also exhibited good activity against stationary phase *B. henselae.* For example, ho wood, which was also extracted from *Cinnamomum* spp. as cinnamon essential oils were shown to be active. Marjoram (sweet), which was extracted from *Origanum* spp. as oregano essential oils were also active. In addition, essential oils extracted from *Citrus* spp. including tangerine, bergamot, lemon, and grapefruit all exhibited strong activity against stationary phase *B. henselae*, and the same for essential oils extracted from *Pelargonium* spp. (geranium bourbon and geranium essential oil) and *Melaleuca* spp. (cajeput and tea tree). Many synergy blend essential oils including “stress relief”, “bandit”, “health shield”, “Tic Tox”, “citrus blast”, and “deep forest” exhibited strong activity against stationary phase *B. henselae* because their effective ingredients were shown to be active by other single essential oils, such as clove, ylang-ylang, lemon, bergamot, grapefruit, cinnamon, oregano, and fir needle. Control drugs including doxycycline (DOX) and azithromycin (AZI) exhibited poor activity against stationary phase *B. henselae* as shown by green (live) cells in fluorescence microscope observation, other antibiotics including gentamicin (GEN), moxifloxacin (MXF), rifampin (RIF), daptomycin (DAP), methylene blue, nitrofurantoin (NIT), and miconazole showed better activity, while not as good as most of the 32 top hits (Figure 1).

### 2.2. MIC Determination of Active Hits

The essential oils listed above were active against the non-growing stationary phase *B. henselae* (Table 1 and Figure 1), and it was necessary to determine the MICs of these active drugs against log-phase growing *B. henselae*. The MICs of essential oils for *B. henselae* were determined by the standard microdilution method, as described in our previous study [27]. As shown in Table 2, cinnamon bark was the most active essential oil among these 32 hits, capable of inhibiting visible *B. henselae* proliferation at the lowest concentration of essential oils tested (0.008%). The health shield, a blend of many active compounds against non-growing *B. henselae* were also highly active against growing *B. henselae*, which could inhibit *B. henselae* proliferation at a concentration of 0.008–0.016%. And the growth of *B. henselae* was efficiently suppressed by bandit, elemi, mountain savory (winter), cedarwood and two oregano essential oils at 0.016–0.032%, and by ylang-ylang, citronella, clove bud, geranium bourbon, allspice, vetiver, cinnamon leaf and geranium essential oil at 0.032–0.063%. Other single essential oils including bergamot, cajeput, marjoram (sweet), fir needle, grapefruit as well as blend essential oils including stress relief, citrus blast, and deep forest were also active with MIC values of 0.063–0.125%. *B. henselae* growing cells were also susceptible to spearmint, tangerine, tea tree, lemon, ho wood, frankincense, and Tic Tox aux huiles essentielles at a concentration of 0.125–0.25%, though relatively higher than others.

### 2.3. Time-Kill Curves of Active Hits

**Figure 2:**
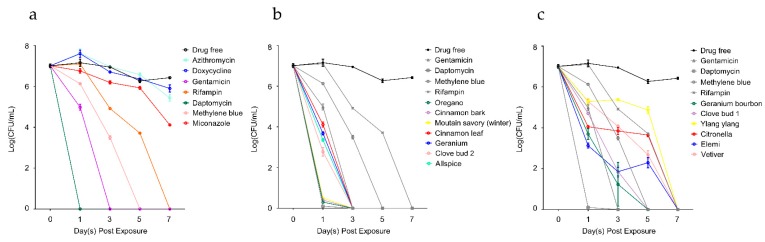
Time-kill curves for essential oils treatment of five-day-old stationary phase *B. henselae* in comparison with control drugs. (**a**) Time-kill curves for control antibiotic treatment. (**b**,**c**) Time-kill curves for essential oil treatment. Drug-free control, daptomycin, gentamicin, methylene blue, and rifampin treatment were the same among a, b, and c. The essential oils or control antibiotics were added to the stationary phase culture respectively at time point 0, and at different times of drug exposure (day 1, day 3, day 5, and day 7), portions of bacteria were removed and washed and plated on Columbia blood agar for CFU counts. The essential oil concentration used in this experiment was 0.032% (*v/v*). Drug concentrations used in this experiment were based on their Cmax and were as follows: 2.4 μg/mL doxycycline, 0.2 μg/mL azithromycin, 10 μg/mL gentamicin, 7.8 μg/mL rifampin, 60 μg/mL daptomycin, 2.9 μg/mL methylene blue, and 6.3 μg/mL miconazole.

Having obtained 32 top hits by primary screens, we performed a time-kill drug exposure assay against a five-day-old stationary phase *B. henselae* culture at a lower concentration of these active essential oils. Here we just selected single essential oil samples for drug exposure assay in order to better evaluate and compare the activity of antimicrobial components among different essential oils. All selected 25 essential oils were applied at 0.032% (*v/v*), respectively. Clinically used antibiotics and the previously identified effective FDA-approved drugs against *B. henselae* were used at their Cmax as controls. As shown in Table 3 and Figure 2b,c, oregano, cinnamon bark, and mountain savory (winter) were the most active essential oils that rapidly killed *B. henselae* with no detectable CFU after one-day exposure. Other active hits, including clove bud 2, allspice, geranium, and cinnamon leaf could eradicate *B. henselae* cells without viable cells being recovered after a three-day drug exposure. Geranium bourbon and clove bud 1 also showed excellent activity which could kill all bacteria by day 5, followed by elemi, vetiver, citronella and ylang ylang that eradicated all *B. henselae* cells by day 7.

As shown in Table 3, grapefruit, tangerine, bergamot, fir needle, frankincense and ho wood were also quite active, reducing 5 log10 CFU/mL after a seven-day exposure. Lemon, as well as marjoram (sweet) also had the capability of killing stationary phase *B. henselae* and reduced the bacterial count by approximately 3 log10 CFU/mL in seven days. However, cajeput, tea tree, cedarwood, and spearmint showed poor activity. Compared with drug-free control, as shown in Figure 2a, some clinically used antibiotics for *Bartonella* treatment, such as azithromycin and doxycycline, had poor activity in killing *B. henselae*, achieving approximately 1 log10 CFU/mL decrease after the seven-day drug exposure. Gentamicin and rifampin showed better activity than azithromycin and doxycycline when used at their Cmax, which could eradicate all *B. henselae* cells respectively by day 3 and day 7. Other FDA-approved drugs effective against *B. henselae* including daptomycin and methylene blue had good activity that led to the eradication of *B. henselae* cells after a one-day or five-day exposure, respectively, while miconazole did not kill all *B. henselae* cells by day 7 when used at Cmax.

### 2.4. Carvacrol and Cinnamaldehyde as Highly Potent Active Ingredient of Essential Oils against Stationary Phase B. henselae

**Figure 3:**
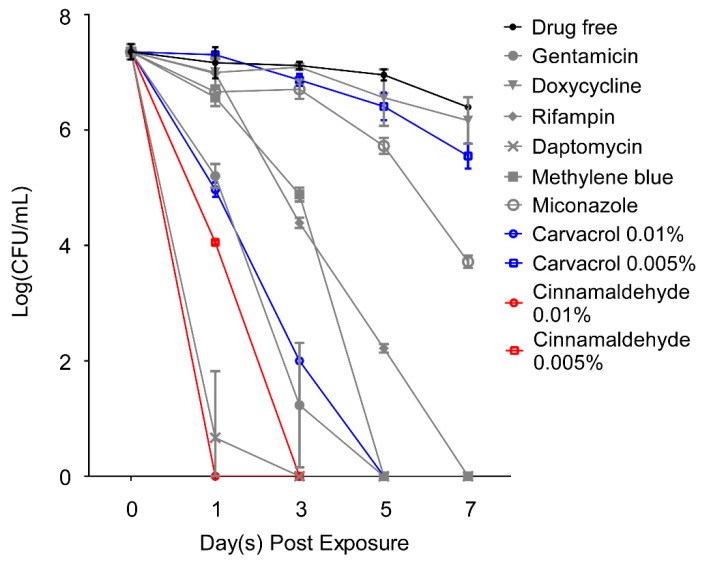
Time-kill curves for carvacrol and cinnamaldehyde treatment of five-day-old stationary phase *B. henselae* in comparison with control drugs. Carvacrol, cinnamaldehyde or control antibiotics were added to the stationary phase culture respectively at time point 0, and at different times of drug exposure (day 1, day 3, day 5, and day 7), portions of bacteria were removed and washed and plated on Columbia blood agar for CFU counts. The concentrations of carvacrol and cinnamaldehyde used were 0.01% (*v/v*) and 0.005% (*v/v*). Drug concentrations used in this experiment were based on their Cmax and were as follows: 2.4 μg/mL doxycycline, 10 μg/mL gentamicin, 7.8 μg/mL rifampin, 60 μg/mL daptomycin, 2.9 μg/mL methylene blue, and 6.3 μg/mL miconazole.

Our previous studies have identified two components, carvacrol and cinnamaldehyde, as highly potent active ingredients of oregano and cinnamon bark essential oils, respectively, which were effective against *B. burgdorferi* [21,22]. As shown above, oregano and cinnamon bark essential oils were also highly active to kill *B. henselae*, so we tested carvacrol and cinnamaldehyde, two major constituents of these two active essential oils, for their antimicrobial activity against *B. henselae*. Carvacrol and cinnamaldehyde were applied at two concentrations, 0.01% (*v/v*) and 0.005% (*v/v*), respectively, for the drug exposure assay against a five-day-old stationary phase *B. henselae* culture. Clinically used antibiotics and the previously identified effective FDA-approved drugs against *B. henselae* were used at their Cmax as controls. As shown in Figure 3, 0.01% carvacrol could eradicate *B. henselae* cells without viable cells being recovered after a five-day drug exposure. Additionally, 0.005% carvacrol led to 2 log10 CFU/mL reduction after a seven-day exposure. Cinnamaldehyde was especially active such that it rapidly killed all stationary phase *B. henselae* cells with no detectable CFU after one-day exposure when used at the concentration of 0.01%, and 0.005% cinnamaldehyde could also eradicate all *B. henselae* cells after a three-day exposure. According to the concentration of original stock, 0.005% carvacrol or cinnamaldehyde was approximately equal to 50 μg/mL. Thus, the antimicrobial activity of carvacrol and cinnamaldehyde against *B. henselae* was comparable to that of effective FDA-approved antibiotics against stationary phase *B. henselae*, including gentamicin, rifampin, daptomycin, and methylene blue, which could eradicate all *B. henselae* cells within the seven-day drug exposure when used at their Cmax.

## 3. Discussion

In this study, we successfully applied the SYBR Green I/PI viability assay for the high-throughput screen of an essential oil collection for activity against stationary phase *B. henselae* as a model of persister drug screens. We identified 32 essential oils at 0.25% concentration which have good activity against stationary phase *B. henselae*. These include four essential oils extracted from plants of genus *Citrus* (tangerine, bergamot, lemon and grapefruit), three from *Origanum* (two oregano essential oils and marjoram), three from *Cinnamomum* (cinnamon bark, cinnamon leaf and ho wood), two from *Pelargonium* (geranium bourbon and geranium essential oil) and two from *Melaleuca* (cajeput and tea tree). Among these 32 top hits, thirteen single essential oils could effectively kill all stationary *B. henselae* cells without CFU detected within a seven-day drug exposure even at a low concentration of 0.032% (*v/v*), where the essential oils of oregano, cinnamon bark, and mountain savory (winter) were the most active ones that eradicated bacteria after a one-day exposure. Some essential oils that showed activity by primary screens exhibited poor activity in drug exposure assay, partly due to the volatility of essential oils during such a long incubation period. Carvacrol and cinnamaldehyde, two active ingredients of effective essential oils, oregano and cinnamon bark, respectively, were shown to be extremely active against stationary phase *B. henselae* that they could eradicate all bacterial cells within a seven-day drug exposure even at a very low concentration ≤ 0.01% (*v/v*). Additionally, the MIC determination showed the 32 active hits were not only active against stationary phase *B. henselae* but also effective in inhibiting the growth of log phase *B. henselae*, especially the essential oil of cinnamon bark.

The pattern that some different plant species of the same genus all possess the antimicrobial ability could serve as a guideline in our future study to obtain more active hits and decode the antimicrobial mechanism. Here, we identified *Citrus*, *Cinnamomum*, *Origanum*, *Pelargonium* and *Melaleuca* as potential genera that might include more plants active against *B. henselae*. *Citrus* plants constitute one of the most valuable and important sources of essential oil served in food processing and medical use. *Citrus limonum* essential oil was reported to have antimicrobial activities with preservative effect against *Listeria monocytogenes* inoculated in minced beef meat [28], and remarkable miticidal activity in vitro and in vivo applications against sarcoptic mange in rabbits [29]. Besides, the essential oil from *Citrus limetta* Risso peels could alleviate skin inflammation, both tested in vitro and in vivo [30], while the essential oil from *Citrus aurantium* L. var. *amara* Engl also had an anti-inflammatory effect [31]. *Citrus* leaf extract was reported to reduce blood pressure and vascular damage in repeatedly heated palm oil diet-induced hypertensive rats [32]. These studies indicated *Citrus* plants could serve in different health care treatments including antimicrobial function. Our study was the first to identify their activity against *B. henselae*.

Previous studies have shown that oregano oil has antibacterial activity against three Gram-positive and two Gram-negative bacteria of their growing log phase [33]. It was also reported to be highly effective against stationary phase *B. burgdorferi* [21]. Cinnamon, clove bud, and allspice were well-known as flavors for food processing, while they were both found to have excellent activity against *B. burgdorferi* stationary phase cells *in vitro*, even better than the persister drug daptomycin [21,22]. Allspice was also known to have antibacterial activities on many organisms [34]. Here, for the first time, we identified essential oils of oregano, cinnamon, clove bud and allspice as having highly potent activities against both the log phase and stationary phase *B. henselae*. It is interesting to note that the high activity of these common essential oils against both *Borrelia* and *Bartonella*, such as oregano, cinnamon bark, and clove bud, indicated that they had the potential to be active against both *Borrelia* and *Bartonella* persistent infections, which clinically may be present as coinfections [35]. However, it is also worth noting that some other essential oils including frankincense, ylang-ylang, fir needle, mountain savory (winter), elemi, and vetiver, are preferentially more active against *B. henselae.* This suggests preferential activity of some essential oils against different bacterial species that possess different cell surface structures, efflux, and physiology.

Other essential oils identified effective in our study have also been proved to have good biological activities by previous studies. It was reported that frankincense and geranium essential oils could suppress tumor progression through the regulation of the AMPK/mTOR pathway in breast cancer [36]. Geranium essential oil could eradicate enterococcal biofilm at a concentration of 150 mg/mL without bacteria developing resistance to it, thus could be a possible alternative to other antimicrobials during endodontic procedures [37]. Frankincense was reported to have anti-inflammatory and antibacterial effects [38]. Mountain savory has been proved to be highly active against methicillin-resistant *Staphylococcus aureus* (MRSA), *Salmonella typhimurium* and *L. monocytogenes* [39,40,41]. According to previous studies, fir honeydew honey had strong antimicrobial activity against *S. aureus, A. baumannii, P. aeruginosa*, *E. coli* and kinds of fungi [42,43,44]. Ylang-ylang products have a wide variety of bioactivities including antimicrobial, antibiofilm, anti-inflammatory, anti-vector, insect-repellent and so on, demonstrating it to be a useful plant to agriculture and medicine [45]. Citronella was reported to have antifungal and antibiofilm activity as well as antimicrobial activity against *Staphylococcus* [46,47]. Extracts from *Canarium* were proved to be active against MRSA and *P. aeruginosa* [48]. Essential oils of vetiver were active against *S. aureus* and showed good antifungal and cytotoxic activities [49,50]. Here, for the first time, we identified the remarkable activity of these essential oils active against both log phase and stationary phase *B. henselae*.

Although our study has identified many active hits from the essential oil collection, future studies are needed to identify the active ingredients of these active essential oils. Here we just tested two active ingredients, carvacrol, and cinnamaldehyde, which showed excellent ability to eradicate stationary phase *B. henselae* even at a much lower concentration than correlated essential oil samples used. Some previous studies have identified the main ingredients of some active essential oils such as mountain savory, thyme, lemongrass, limette, and cumin, including geranial, β-pinene, thymol, γ-terpinene, citronellal and so on [51]. The antimicrobial activity of these components should be studied thoroughly in the future in order to determine their utility.

It is worth noticing that clinically used drugs for bartonellosis treatment including doxycycline, azithromycin, rifampin, moxifloxacin, and gentamicin showed poor capability in eradicating stationary phase *B. henselae* cells (Table 1 and Figure 1), as they mainly target growing cells in log phase. The discrepant efficacies of antibiotics between in vitro MIC data and clinical data from patients with bartonellosis were also reported and the poor activities of current clinically used antibiotics against stationary phase *B. henselae* as shown in our study could partly explain for the treatment failure and persistence of infection [11]. The microscopic observation showed that stationary phase *B. henselae* cells tended to aggregate together (Figure 1), which might protect them from attack by antimicrobial agents. The bacterial cell membrane is a known target of some persister drugs, and it is interesting to note that essential oils are exactly agents targeting the membrane because of their lipophilicity. Due to this property, essential oils could exhibit much higher activity against stationary phase *B. henselae* in aggregated biofilm form than current clinically used antibiotics for *Bartonella* infections and could be considered promising candidates for further evaluation. However, it should also be noted that the high lipophilicity of essential oils might cause *B. henselae* cells or biofilm structures to be dissolved, leading to a reduction of the dead cell number and as a result, the residual viability percentage by the SYBR Green I/PI assay might be misinterpreted. It is also worth noting that as DMSO could permeabilize the bacterial membranes, it could increase the antibacterial effect of the essential oils by increasing the bacterial susceptibility to lipophilic compounds.

Another promising strategy for developing a more effective treatment for bartonellosis is the drug combination of active essential oils with antibiotics to avoid resistance development and improve the efficacy of the treatment. Future studies are needed to evaluate drug combinations of two or more newly identified essential oil candidates with current clinically used drugs in combination, in order to better target diverse bacterial populations of different phases or forms that can happen in the host as indicated by the Yin-Yang model [14]. There were some previous studies of evaluating the antimicrobial activity of combined essential oil samples against multidrug resistance (MDR) *E. coli*, *K. pneumoniae*, MRSA, *S. epidermidis*, *Propionibacterium acnes*, as well as airborne bacteria and fungi in hospital rooms, and some of the essential oil combinations did have better activity than used alone [52,53,54].

In this study, we identified a range of essential oils with high activity against stationary phase *B. henselae* in vitro. Because *B. henselae* can reside and propagate inside erythrocytes and/or endothelial cells in humans and animals [55,56], which could provide a shelter that protects them from the host immune responses and exposure to antibiotics, future studies are needed to evaluate the activities of selected essential oils against intracellular *B. henselae*. The active ingredients of many effective essential oils remain unknown, and it will take substantial effort to characterize and identify the active components, which is beyond the scope of the current study. These should be studied thoroughly in the future in order to identify the active components, decode the antimicrobial mechanism, and further evaluate their activity in vivo. We are fully aware that while the number of active components of essential oils may be subject to variations from different batches or sources, just like any natural products including essential oils, this should not change the overall findings or conclusions of the study. Further pharmacokinetic study is required to test whether it is possible to achieve effective concentrations in vivo. Further validation using appropriate animal models of bartonellosis is required to assess the safety and efficacy of identified essential oils in vivo. As far as we know, the effective concentration of two highly active ingredients, carvacrol, and cinnamaldehyde, was comparable to the Cmax of some antibiotics, which could serve as promising drug candidates that may achieve efficacy when used in vivo. Our study was performed with *B. henselae* and future studies are needed to test if the findings here apply to other *B. henselae* strains and also closely related pathogenic *Bartonella* species, such as *B. quintana* and *B. bacilliformis*.

## 4. Materials and Methods

### 4.1. Bacterial Strain, Culture Media and Culture Conditions

The *Bartonella henselae* JK53 strain was obtained from BEI Resources (ATCC). Based on the culture medium developed in a previous study [57], *B. henselae* JK53 was cultured in Schneider’s Drosophila medium (Life Technologies Limited, Paisley, UK) supplemented with 10% fetal bovine serum (FBS) (Sigma-Aldrich, Co., St. Louis, MO, USA) and 5% sucrose (Fisher Scientific, New Jersey, USA) in microaerophilic incubator without shaking at 37℃, 5% CO_2_. As previously measured [24], *B. henselae* JK53 rapidly went into the logarithmic growth phase and reached a growth peak after two days under such culture conditions. The one-day-old and five-day-old culture were considered as log phase and stationary phase, respectively. The Columbia anaerobic sheep blood agar (HemoStat Laboratories, Dixon, CA, USA) was used to perform the drug exposure assay, which was also cultured at 37 °C, 5% CO_2_.

### 4.2. Drugs, Essential Oils and their Active Ingredients

A panel of 149 essential oils was purchased from Plant Therapy (ID, USA), Natural Acres (MO, USA), or Plant Guru (NJ, USA). Detailed information, as well as some GC-MS reports of these essential oils, are available at the vendors’ websites. The main chemical compositions of active essential oils were summarized in Table S1 based on vendors’ GC-MS reports or previous studies. Carvacrol and cinnamaldehyde were purchased from Sigma-Aldrich (USA). DMSO-soluble essential oils and carvacrol and cinnamaldehyde were dissolved in dimethyl sulfoxide (DMSO) at 5% (*v/v*), followed by dilution at 1:10 into five-day-old stationary bacteria cultures to achieve 0.5% final concentration. To make further dilutions for evaluating anti-*Bartonella* activity, the 0.5% essential oil treatments were further diluted in the same stationary bacteria cultures to achieve desired concentrations. DMSO-insoluble essential oils were added directly to five-day-old stationary bacteria cultures to form emulsion by adequate vortexing, followed by immediate transfer of the emulsion into the same stationary cultures to make serial dilutions to achieve desired final concentrations. Antibiotics including azithromycin (AZI), daptomycin (DAP), doxycycline (DOX), gentamicin (GEN), methylene blue, miconazole, moxifloxacin (MXF), nitrofurantoin (NIT), rifampin (RIF) were purchased from Sigma-Aldrich and were dissolved in appropriate solvents [58] to form 10mg/mL or 100mM stock solutions. All the antibiotic stocks were filter-sterilized by 0.2 μm filter except the DMSO stocks and then diluted and stored at −20 °C.

### 4.3. Microscopy Techniques

Drug-treated or control *B. henselae* JK53 cell suspensions were examined using BZ-X710 All-in-One fluorescence microscope (KEYENCE, Inc.), with SYBR Green I (100 × stock, Invitrogen) and propidium iodide (PI, 600 μM, Sigma) mixture used for staining. The SYBR Green I/PI dye was added to the sample at 1:10 dilution and mixed thoroughly to assess the bacterial viability by using the ratio of green/red fluorescence to determine the residual viability percentage, respectively, as described previously [20]. The residual bacteria viability was confirmed by analyzing three representative images of the same bacterial cell suspension using fluorescence microscopy. BZ-X Analyzer and Image Pro-Plus software were used to quantitatively determine the fluorescence intensity.

### 4.4. Screening of Essential Oil Library against Stationary Phase B. Henselae JK53

For the high-throughput essential oil screening, each essential oil was assayed in two concentrations, 0.5% (*v/v*) and 0.25% (*v/v*). Firstly, 20 μL 5% essential oil DMSO stocks or emulsion were added to the 96-well plate containing 180 μL of the five-day-old stationary phase *B. henselae* JK53 culture to obtain the desired concentration of 0.5%. Then, the 0.25% treatment concentration was obtained by mixing 100 μL 0.5% treatment with 100 μL five-day-old *B. henselae* JK53 culture. Antibiotics including AZI, DAP, DOX, GEN, methylene blue, miconazole, MXF, NIT, and RIF were used as control drugs at 20 μM. Plates were sealed and placed in a 37 °C incubator without shaking over a period of three days. SYBR Green I/ PI viability assay was used to assess the live and dead cells after drug exposure as described [20]. Briefly, the SYBR Green I/PI dye was added to the sample at 1:10 dilution and mixed thoroughly. The plates were incubated at room temperature in the dark for 15 min followed by plate reading using a microplate reader (HTS 7000 plus Bioassay Reader, PerkinElmer Inc., USA). The green/red (538 nm/650 nm) fluorescence ratio of each well was used for calculating the residual viability percentage. With the least-square fitting analysis, the regression equation and regression curve of the relationship between residual viability percentage and green/red fluorescence ratio was obtained, which was used in the calculation as described previously [24]. All tests were run in triplicate.

### 4.5. Drug Exposure Assay

Based on primary screening results, active hits were further confirmed by drug exposure assay. The 5-day-old *B. henselae* JK53 stationary phase culture was used for drug exposure experiments, which was treated with 0.032% (*v/v*) active essential oils respectively. Then two highly potent active ingredients, carvacrol, and cinnamaldehyde, of active essential oils oregano and cinnamon bark, respectively, were also tested by drug exposure assay at a very low concentration of 0.01% (*v/v*) and 0.005% (*v/v*). Control antibiotics were used at their Cmax. The drug exposure assay was carried out in 15 mL Eppendorf tubes over the course of seven days at 37 °C, 5% CO_2_ without shaking. At each time point we measured, the bacteria cells were collected by centrifugation and rinsed twice with fresh Schneider’s medium followed by resuspension in fresh Schneider’s medium. Then the cell suspension was serially diluted and each dilution was plated on Columbia blood agar plates for viable bacterial counts (colony forming unit, CFU). All tests were run in triplicate.

### 4.6. MIC Determination

The standard microdilution method was used to measure the minimum inhibitory concentration (MIC) needed to inhibit the visible growth of *B. henselae* JK53 after a five-day incubation period. The diluted one-day-old *B. henselae* JK53 logarithmic phase culture was used for MIC determination. 1×10^6^ bacteria cells were inoculated into the well of the 96-well plate containing 180 μL fresh modified Schneider’s medium per well. Then 20 μL 5% essential oil stocks were added into each well respectively to achieve 0.5% final concentration. Other lower concentrations were obtained by mixing 0.5% treatment with diluted one-day-old *B. henselae* JK53 logarithmic phase culture. Plates were sealed and incubated at 37 °C without shaking for five days. Then the bacteria cell proliferation was assessed using the SYBR Green I/PI assay and the bacterial counting chamber after the incubation. All tests were run in triplicate.

## 5. Conclusions

In summary, this is the first study of a high throughput drug screen against stationary phase *B. henselae* using a collection of essential oils where we identified a range of highly active essential oils. Two active ingredients of these effective hits, carvacrol, and cinnamaldehyde were also identified to have strong antimicrobial activity against stationary phase *B. henselae*, while other ingredients still need to be identified and evaluated thoroughly for both efficacy and toxicity. Future studies are needed to determine if essential oil candidates are more effective against *Bartonella* persisters as well as biofilm bacteria in combination with antibiotics in vitro and in animal models of *Bartonella* infections.

## Figures and Tables

**Table 1:** Activity of top 32 active hits that had good activity against stationary phase *B. henselae* ^1^.

Essential Oils and Control Drugs	Plant or Ingredients of Essential Oils	Residual Viability (%) after 0.5% EO or 20 μM Antibiotic Treatment		Residual Viability (%) after 0.25% EO Treatment	
		Plate Reader ^2^	Microscope ^3^	Plate Reader ^2^	Microscope ^3^
Drug free control		74%	74%		
Doxycycline		26%	57%		
Gentamicin		9%	35%		
Moxifloxacin		22%	40%		
Azithromycin		23%	67%		
Rifampin		25%	44%		
Daptomycin		8%	18%		
Methylene Blue		16%	27%		
Nitrofurantoin		18%	50%		
Miconazole		19%	44%		
Frankincense	*Boswellia serrata*	5%	11%	6%	10%
Ylang ylang	*Cananga odorata*	5%	9%	8%	10%
Tangerine	*Citrus reticulata*	6%	6%	5%	12%
Bergamot	*Citrus bergamia*	6%	18%	10%	15%
Marjoram (sweet)	*Origanum majorana*	6%	13%	5%	15%
Cajeput	*Melaleuca cajeputi*	7%	21%	9%	21%
Lemon	*Citrus limonum*	7%	10%	4%	11%
Oregano	*Origanum vulgare* *hirtum*	7%	7%	7%	20%
Geranium bourbon	*Pelargonium graveolens*	8%	20%	11%	22%
Tea tree	*Melaleuca alternifolia*	8%	12%	5%	25%
Fir needle	*Abies siberica*	8%	25%	10%	26%
Stress relief	synergy blend of essential oils of bergamot, patchouli, sweet orange, ylang ylang, pink grapefruit, gurjum	8%	15%	6%	12%
Mountain savory (winter)	*Satureja montana*	8%	25%	21%	32%
Bandit	synergy blend of essential oils of clove, cinnamon, lemon, rosemary, eucalyptus	8%	8%	12%	20%
Cinnamon leaf	*Cinnamomum zeylanicum*	8%	35%	10%	25%
Citronella	*Cymbopogon winterianus*	8%	15%	12%	23%
Health shield	blend of cinnamon, clove, eucalyptus, lemon and rosemary oils	9%	18%	17%	20%
Spearmint	*Mentha spicata*	9%	9%	4%	20%
Ho wood	*Cinnamomum camphora*	9%	20%	11%	29%
Tic Tox aux huiles essentielles	blend of essential oils of savory, sage officinale, wild chamomile, clove, compact oregano, cinnamon and niaouli	11%	21%	14%	14%
Citrus blast	synergy blend of *Citrus sinesis, Citrus limonum, Citrus reticulata blanco var tangerina, Citrus bergamia, Citrus reticulata, Citrus clementina, Vanilla planifolia*	11%	13%	11%	30%
Elemi	*Canarium luzonicum*	12%	25%	14%	32%
Vetiver	*Vetiveria zizanoides*	12%	26%	8%	18%
Grapefruit	*Citrus paradisi*	12%	35%	11%	36%
Clove bud 1	*Eugenia caryophyllata*	13%	36%	9%	23%
Deep forest	synergy blend of *Abies sibirica ledeb, Abies alba, Pinus sylvestris, Cupressus sempervirens,* *Cedrus deodora*	13%	20%	12%	50%
Geranium	*Pelargonium asperum*	14%	23%	15%	20%
Clove bud 2	*Syzygium aromaticum L*	15%	15%	14%	18%
Oil of oregano	*Origanum vulgare* *hirtum*	15%	52%	19%	55%
Allspice	*Pimenta officinalis*	16%	35%	6%	30%
Cedarwood	*Cedrus deodora*	17%	53%	10%	23%
Cinnamon bark	*Cinnamomum zeylanicum*	18%	40%	13%	45%

^1^ A five-day-old stationary phase *B. henselae* culture was treated with essential oils (0.5%)(*v/v*) or control drugs (20 μM) for three days. ^2^ Residual viability was calculated according to the regression equation and the ratio of Green/Red fluorescence obtained by SYBR Green I/PI assay. ^3^ Residual viability was assayed by fluorescence microscope counting.

**Table 2:** Minimal inhibitory concentrations (MICs) of top hit essential oils against *B. henselae* ^1^.

Essential Oils	Plant or Ingredients of Essential Oils	MIC (*v/v*)
Cinnamon bark	*Cinnamomum zeylanicum*	<0.008%
Health shield	blend of cinnamon, clove, eucalyptus, lemon and rosemary oils	0.008–0.016%
Bandit	synergy blend of essential oils of clove, cinnamon, lemon, rosemary, eucalyptus	0.016–0.032%
Oregano	*Origanum vulgare hirtum*	0.016–0.032%
Elemi	*Canarium luzonicum*	0.016–0.032%
Oil of oregano	*Origanum vulgare hirtum*	0.016–0.032%
Mountain savory (winter)	*Satureja montana*	0.016–0.032%
Cedarwood	*Cedrus deodora*	0.016–0.032%
Ylang ylang	*Cananga odorata*	0.032–0.063%
Citronella	*Cymbopogon winterianus*	0.032–0.063%
Clove bud 1	*Eugenia caryophyllata*	0.032–0.063%
Clove bud 2	*Syzygium aromaticum L*	0.032–0.063%
Geranium bourbon	*Pelargonium graveolens*	0.032–0.063%
Allspice	*Pimenta officinalis*	0.032–0.063%
Vetiver	*Vetiveria zizanoides*	0.032–0.063%
Cinnamon leaf	*Cinnamomum zeylanicum*	0.032–0.063%
Geranium	*Pelargonium asperum*	0.032–0.063%
Stress relief	synergy blend of essential oils of bergamot, patchouli, sweet orange, ylang ylang, pink grapefruit, gurjum	0.063–0.125%
Bergamot	*Citrus bergamia*	0.063–0.125%
Cajeput	*Melaleuca cajeputi*	0.063–0.125%
Marjoram (sweet)	*Origanum majorana*	0.063–0.125%
Citrus blast	synergy blend of essential oils of bergamot, patchouli, sweet orange, ylang ylang, pink grapefruit, gurjum	0.063–0.125%
Deep forest	synergy blend of *Abies sibirica ledeb, Abies alba, Pinus sylvestris, Cupressus sempervirens, Cedrus deodora*	0.063–0.125%
Fir needle	*Abies siberica*	0.063–0.125%
Grapefruit	*Citrus paradisi*	0.063–0.125%
Spearmint	*Mentha spicata*	0.125–0.25%
Tangerine	*Citrus reticulata*	0.125–0.25%
Tea tree	*Melaleuca alternifolia*	0.125–0.25%
Lemon	*Citrus limonum*	0.125–0.25%
Ho wood	*Cinnamomum camphora*	0.125–0.25%
Frankincense	*Boswellia serrata*	0.125–0.25%
Tic Tox aux huiles essentielles	blend of essential oils of savory, sage officinale, wild chamomile, clove, compact oregano, cinnamon and niaouli	0.125–0.25%

^1^ The MIC testing for *B. henselae* was set up as described in Methods.

**Table 3:** Drug exposure assay of selected active essential oils against a five-day-old stationary phase *B. henselae* culture ^1^.

Essential Oils and Control Drugs ^2^	CFU/mL after Drug Exposure			
	1 Day	3 Day	5 Day	7 Day
Drug free control	1.50 ± 0.53 × 10^7^	8.83 ± 0.29 × 10^6^	1.88 ± 0.40 × 10^6^	2.67 ± 0.29 × 10^6^
Doxycycline	4.17 ± 1.44 × 10^7^	5.07 ± 0.38 × 10^6^	2.30 ± 0.10 × 10^6^	8.33 ± 2.89 × 10^5^
Azithromycin	4.50 ± 2.00 × 10^7^	9.17 ± 0.29 × 10^6^	3.80 ± 0.72 × 10^6^	2.83 ± 1.04 × 10^5^
Gentamicin	9.83 ± 2.93 × 10^4^	0	0	0
Rifampin	1.27 ± 0.15 × 10^7^	8.33 ± 0.76 × 10^4^	5.17 ± 0.29 × 10^3^	0
Daptomycin	0	0	0	0
Methylene blue	1.35 ± 0.13 × 10^6^	3.17 ± 0.58 × 10^3^	0	0
Miconazole	5.83 ± 1.53 × 10^6^	1.57 ± 0.28 × 10^6^	8.50 ± 1.32 × 10^5^	1.30 ± 0.10 × 10^4^
Oregano	0	0	0	0
Cinnamon bark	0	0	0	0
Mountain savory (winter)	0	0	0	0
Clove bud 2	6.50 ± 3.46 × 10^2^	0	0	0
Allspice	2.27 ± 0.33 × 10^3^	0	0	0
Geranium	4.83 ± 0.76 × 10^3^	0	0	0
Cinnamon leaf	1.33 ± 0.35 × 10^4^	0	0	0
Geranium bourbon	5.50 ± 2.65 × 10^3^	5.00 ± 5.00 × 10	0	0
Clove bud 1	5.00 ± 0.00 × 10^4^	8.33 ± 5.77 × 10	0	0
Elemi	1.38 ± 0.42 × 10^3^	5.00 ± 5.00 × 10	2.17 ± 1.04 × 10^2^	0
Vetiver	2.00 ± 0.50 × 10^5^	1.18 ± 0.19 × 10^4^	5.17 ± 2.47 × 10^2^	0
Citronella	1.13 ± 0.12 × 10^4^	7.33 ± 2.84 × 10^3^	4.50 ± 0.87 × 10^3^	0
Ylang ylang	2.00 ± 0.87 × 10^5^	2.38 ± 0.19 × 10^5^	7.83 ± 3.01 × 10^4^	0
Grapefruit	1.02 ± 0.19 × 10^4^	3.17 ± 1.89 × 10^4^	5.33 ± 1.26 × 10^3^	6.67 ± 5.77 × 10
Tangerine	3.17 ± 0.29 × 10^4^	2.08 ± 0.58 × 10^4^	4.50 ± 2.29 × 10^3^	6.67 ± 5.77 × 10
Bergamot	8.17 ± 2.25 × 10^3^	2.62 ± 0.35 × 10^4^	6.83 ± 0.76 × 10^3^	1.67 ± 0.58 × 10^2^
Fir needle	4.17 ± 1.61 × 10^3^	2.32 ± 0.41 × 10^4^	1.10 ± 0.13 × 10^4^	1.67 ± 0.58 × 10^2^
Frankincense	1.35 ± 0.22 × 10^5^	8.17 ± 1.53 × 10^5^	1.48 ± 0.29 × 10^6^	1.83 ± 0.76 × 10^2^
Ho wood	5.00 ± 0.50 × 10^6^	7.50 ± 2.65 × 10^5^	1.37 ± 0.28 × 10^5^	4.17 ± 1.44 × 10^2^
Lemon	3.17 ± 1.15 × 10^4^	1.03 ± 0.28 × 10^5^	8.67 ± 0.76 × 10^4^	4.33 ± 2.31 × 10^3^
Marjoram (Sweet)	2.17 ± 1.53 × 10^5^	2.13 ± 0.28 × 10^6^	2.22 ± 0.25 × 10^6^	7.50 ± 1.32 × 10^3^
Cajeput	2.50 ± 0.87 × 10^6^	9.43 ± 0.40 × 10^6^	3.20 ± 0.26 × 10^6^	1.62 ± 0.25 × 10^5^
Tea tree	8.00 ± 2.18 × 10^5^	9.33 ± 0.29 × 10^6^	3.97 ± 0.45 × 10^6^	3.17 ± 0.76 × 10^6^
Cedarwood	2.33 ± 2.31 × 10^5^	2.73 ± 0.33 × 10^6^	3.40 ± 0.36 × 10^6^	3.52 ± 0.18 × 10^6^
Spearmint	4.33 ± 1.26 × 10^5^	9.17 ± 0.29 × 10^6^	3.67 ± 0.58 × 10^6^	3.68 ± 0.38 × 10^6^

^1^ A five-day-old stationary phase *B. henselae* culture was treated with essential oils or control drugs. The beginning CFU for the five-day-old stationary phase *B. henselae* culture was about 1 × 10^7^ CFU/mL. At different times of drug exposure (day 1, day 3, day 5, and day 7), portions of bacteria were removed, washed, and plated on Columbia blood agar for CFU counts. ^2^ The essential oil concentration used in this experiment was 0.032% (*v/v*). Drug concentrations used in this experiment were based on their Cmax and were as follows: 2.4 μg/mL doxycycline, 0.2 μg/mL azithromycin, 10 μg/mL gentamicin, 7.8 μg/mL rifampin, 60 μg/mL daptomycin, 2.9 μg/mL methylene blue, and 6.3 μg/mL miconazole.

## Supplementary Materials

The following is available online at https://www.mdpi.com/2079-6382/8/4/246/s1, Table S1: Chemical compositions of top hit essential oils against *B. henselae*.
