# Supplementary material for: Essential Oils with High Activity against Stationary Phase Bartonella henselae
Source: Antibiotics (Basel). 2019 Nov 30;8(4):246. doi: 10.3390/antibiotics8040246 (PMC6963529; doi:10.3390/antibiotics8040246)
Supplement: Supplementary file 1 [file antibiotics-08-00246-s001.pdf]

Table S1. Chemical compositions of top hit essential oils against *B. henselae*.

Essential oils	Plant of essential oils	Plant part	Main chemical composition	Reference
Cinnamon bark	*Cinnamomum zeylanicum*	bark	cinnamaldehyde, camphene, 1,8-cineole, α-terpineol, bornyl acetate, etc.	[1]
Oregano	*Origanum vulgare hirtum*	herbs	thymol, γ-terpinene, carvacrol, p-cymene, myrcene, etc.	[2]
Elemi	*Canarium luzonicum*	resin	elemol, elemecin, sabinene, α-phellandrene, p-cymene, limonene, α-terpineol, etc.	[3]
Mountain savory (winter)	*Satureja montana*	herbs	carvacrol, borneol, carvacrylacetate, α-terpineol, thymol, etc.	[4]
Cedarwood	*Cedrus deodora*	wood	wikstromol, matairesinol, benzylbutyrolactol, BDFD	[5]
Ylang ylang	*Cananga odorata*	flowers	linalool, β-caryophyllene, α-humulene, γ-muurolene, germacrene D, (3E,6E)-α-farnesene, δ-cadinene, benzyl benzoate	[6]
Citronella	*Cymbopogon winterianus*	leaves	citronellal, isomenthone, citronellol, geraniol, limonene, etc.	[7]
Clove bud 1	*Eugenia caryophyllata*	bud	eugenol, eugenyl acetate, β-caryophyllene, carvacrol, cinnamaldehyde, thymol, etc.	[8]
Clove bud 2	*Syzygium aromaticum L*	bud	eugenol, β-caryophyllene, eugenyl acetate, etc.	[9]
Geranium bourbon	*Pelargonium graveolens*	leaves and flowers	linalool, citronellol, iso-menthone, geraniol, citronellyl formate, geraniol formate, etc.	[10]
Allspice	*Pimenta officinalis*	berries	eugenol, methyleugenol, β-caryophyllene, α-humulene, etc.	[11]
Vetiver	*Vetiveria zizanoides*	root	cycloisolongifolene, khusimene, β-cadinene, β-guaiene, β-vetivenene, etc.	[12]
Cinnamon leaf	*Cinnamomum zeylanicum*	leaf	eugenol, benzyl benzoate, linalool, cinnamaldehyde, etc.	[13]
Geranium	*Pelargonium asperum*	herbs	citronellol, citronellyl ester, geraniol, buthyl anthranilate, etc.	[14]
Bergamot	*Citrus bergamia*	fruit peel	limonene, β-pinene, γ-terpinene, linalool, linalyl acetate, etc.	[15]
Cajeput	*Melaleuca cajeputi*	leaves and buds	ethanone, 4H-1-benzopyran-4-one, 1,4-naphthalenedione, naphthalene, etc.	[16]

Marjoram (sweet)	*Origanum majorana*	leaves and flowers	α-terpineol, (-)-Terpinen-4-ol, 5-Isopropyl-2-methylbicyclo[3.1.0]hexan-2-ol, β-cymene, β-phellandrene, etc.	[17]
Fir needle	*Abies siberica*	needles	α-pinene, camphene, β-pinene, Δ3-carene, limonene, bornyl acetate, etc.	[18]
Grapefruit	*Citrus paradisi*	peel	limonene, β-myrcene, α-pinene, etc.	[19]
Spearmint	*Mentha spicata*	flowering herbs	carvone, limonene, 1,8-cineole, β-caryophyllene, germacrene D, etc.	[20]
Tangerine	*Citrus reticulata*	fruit peel	citronellal, thymol, geranyl acetate, β-elemen, germacrene B, etc.	[21]
Tea tree	*Melaleuca alternifolia*	leaves	α-pinene, α-terpinene, 1,8-cineole, γ-terpinene, terpinolene, terpinen-4-ol, α-terpineol, etc.	[22]
Lemon	*Citrus limonum*	peel	β-pinene, limonene, linalool, α-terpineol, linalyl acetate, nerolidol, farnesol, etc.	[23]
Ho wood	*Cinnamomum camphora*	twigs and bark	D-camphor, 1,8-cineole, α-terpineol, while D-camphor, linalool, 1,8-cineole, etc.	[24]
Frankincense	*Boswellia serrata*	resin	α-pinene, α-thujene, methylchavicol, sabinene, methyleugenol, myrcene, limonene, p-cymene, etc.	[25]

References

1. Falleh, H.; Ben Jemaa, M.; Djebali, K.; Abid, S.; Saada, M.; Ksouri, R. Application of the mixture design for optimum antimicrobial activity: Combined treatment of Syzygium aromaticum, Cinnamomum zeylanicum, Myrtus communis, and Lavandula stoechas essential oils against Escherichia coli. *J. Food. Process. Preserv*. **2019**, 00: e14257.
2. Vinciguerra, V.; Rojas, F.; Tedesco, V.; Giusiano, G.; Angiolella, L. Chemical characterization and antifungal activity of Origanum vulgare, Thymus vulgaris essential oils and carvacrol against Malassezia furfur. *Nat. Prod. Res*. **2019**, 33, 3273-3277.
3. Nikolic, M.; Smiljkovic, M.; Markovic, T.; Cirica, A.; Glamoclija, J.; Markovic, D.; Sokovic, M. Sensitivity of clinical isolates of Candida to essential oils from Burseraceae family. *EXCLI J*. **2016**, 15, 280-289.
4. Santos, J.D.C.; Coelho, E.; Silva, R.; Passos, C.P.; Teixeira, P.; Henriques, I.; Coimbra, M.A. Chemical composition and antimicrobial activity of Satureja montana byproducts essential oils. *Industrial Crops and Products*. **2019**, 137, 541-548.
5. Dhayabaran, D.; Florance, E.J.; Nandakumar, K.; Shanmugarathinam, A.; Puratchikody, A. Anticonvulsant activity of fraction isolated from ethanolic extract of heartwood of Cedrus deodara. *J. Nat. Med*. **2014**, 68, 310-315.
6. Compositions of essential oils Available online: https://www.theplantguru.com/amfile/file/download/file_id/28/product_id/734/ (accessed on 8 November 2019)
7. Compositions of essential oils Available online: https://www.theplantguru.com/amfile/file/download/file_id/9/product_id/714/ (accessed on 8 November 2019)
8. Chaieb, K.; Hajlaoui, H.; Zmantar, T.; Kahla-Nakbi, A.B.; Rouabhia, M.; Mahdouani, K.; Bakhrouf, A. The chemical composition and biological activity of clove essential oil, Eugenia caryophyllata (Syzigium aromaticum L. Myrtaceae): a short review. *Phytother. Res*. **2007**, 21, 501-506.

9. Compositions of essential oils Available online: https://www.theplantguru.com/amfile/file/download/file_id/11/product_id/726/ (accessed on 8 November 2019)
10. Essid, R.; Hammami, M.; Gharbi, D.; Karkouch, I.; Hamouda, T.B.; Elkahoui, S.; Limam, F.; Tabbene, O. Antifungal mechanism of the combination of Cinnamomum verum and Pelargonium graveolens essential oils with fluconazole against pathogenic Candida strains. *Appl. Microbiol. Biotechnol*. **2017**, 101, 6993-7006.
11. Compositions of essential oils Available online: https://www.theplantguru.com/amfile/file/download/file_id/1/product_id/622/ (accessed on 8 November 2019)
12. David, A.; Wang, F.; Sun, X.; Li, H.; Lin, J.; Li, P.; Deng, G. Chemical Composition, Antioxidant, and Antimicrobial Activities of Vetiveria zizanioides (L.) Nash Essential Oil Extracted by Carbon Dioxide Expanded Ethanol. *Molecules*. **2019**, 24.
13. Dongmo, P.M.J.; Tatsadjieu, L.N.; Tchoumbougnang, F.; Sameza, M.L.; Dongmo, B.N.; Zollo, P.H.A.; Menut, C. Chemical Composition, Antiradical and Antifungal Activities of Essential Oil of the Leaves of Cinnamomum Zeylanicum Blume from Cameroon. *Natural Product Communications*. **2007**, 2.
14. Ouedrhiri, W.; Balouiri, M.; Bouhdid, S.; Harki, E.H.; Moja, S.; Greche, H. Antioxidant and antibacterial activities of Pelargonium asperum and Ormenis mixta essential oils and their synergistic antibacterial effect. *Environ. Sci. Pollut. Res. Int*. **2018**, 25, 29860-29867.
15. Al-Abd, N.M.; Mohamed Nor, Z.; Mansor, M.; Azhar, F.; Hasan, M.S.; Kassim, M. Antioxidant, antibacterial activity, and phytochemical characterization of Melaleuca cajuputi extract. *BMC. Complement. Altern. Med*. **2015**, 15, 385.
16. Compositions of essential oils Available online: https://www.theplantguru.com/amfile/file/download/file_id/5/product_id/646/ (accessed on 8 November 2019)
17. Makrane, H.; Aziz, M.; Berrabah, M.; Mekhfi, H.; Ziyyat, A.; Bnouham, M.; Legssyer, A.; Elombo, F.K.; Gressier, B.; Eto, B. Myorelaxant Activity of essential oil from Origanum majorana L. on rat and rabbit. *J. Ethnopharmacol*. **2019**, 228, 40-49.
18. Ou, M.C.; Liu, Y.H.; Sun, Y.W.; Chan, C.F. The Composition, Antioxidant and Antibacterial Activities of Cold-Pressed and Distilled Essential Oils of Citrus paradisi and Citrus grandis (L.) Osbeck. *Evid. Based. Complement. Alternat. Med*. **2015**, 2015, 804091.
19. Compositions of essential oils Available online: https://www.theplantguru.com/amfile/file/download/file_id/15/product_id/810/ (accessed on 8 November 2019)
20. Bardaweel, S.K.; Bakchiche, B.; Ha, A.L.; Rezzoug, M.; Gherib, A.; Flamini, G. Chemical composition, antioxidant, antimicrobial and Antiproliferative activities of essential oil of Mentha spicata L. (Lamiaceae) from Algerian Saharan atlas. *BMC. Complement. Altern. Med*. **2018**, 18, 201.
21. Duan, L.; Guo, L.; Dou, L.L.; Zhou, C.L.; Xu, F.G.; Zheng, G.D.; Li, P.; Liu, E.H. Discrimination of Citrus reticulata Blanco and Citrus reticulata 'Chachi' by gas chromatograph-mass spectrometry based metabolomics approach. *Food. Chem*. **2016**, 212, 123-127.
22. Ben Hsouna, A.; Ben Halima, N.; Smaoui, S.; Hamdi, N. Citrus lemon essential oil: chemical composition, antioxidant and antimicrobial activities with its preservative effect against Listeria monocytogenes inoculated in minced beef meat. *Lipids. Health. Dis*. **2017**, 16, 146.
23. Compositions of essential oils Available online: https://www.theplantguru.com/amfile/file/download/file_id/26/product_id/998/ (accessed on 8 November 2019)
24. Guo, S.; Geng, Z.; Zhang, W.; Liang, J.; Wang, C.; Deng, Z.; Du, S. The Chemical Composition of Essential Oils from Cinnamomum camphora and Their Insecticidal Activity against the Stored Product Pests. *Int. J. Mol. Sci*. **2016**, 17.
25. Al-Yasiry, A.R.; Kiczorowska, B. Frankincense--therapeutic properties. *Postepy. Hig. Med. Dosw (Online)*. **2016**, 70, 380-391.
